# African Swine Fever Virus Exhibits Distinct Replication Defects in Different Cell Types

**DOI:** 10.3390/v14122642

**Published:** 2022-11-26

**Authors:** Yanni Gao, Tingting Xia, Juan Bai, Lujie Zhang, Xiaolin Jiang, Xing Yang, Keshan Zhang, Ping Jiang

**Affiliations:** 1 Key Laboratory of Animal Diseases Diagnostic and Immunology, Ministry of Agriculture, MOE Joint International Research Laboratory of Animal Health and Food Safety, College of Veterinary Medicine, Nanjing Agricultural University, Nanjing 210095, China; 2 State Key Laboratory of Veterinary Etiological Biology, National Foot and Mouth Disease Reference Laboratory, Lanzhou Veterinary Research Institute, Chinese Academy of Agricultural Sciences, Lanzhou 730046, China; 3 Jiangsu Co-Innovation Center for the Prevention and Control of Important Animal Infectious Diseases and Zoonoses, Yangzhou University, Yangzhou 225009, China

**Keywords:** African swine fever virus, virus replication, stress granules, protein translation

## Abstract

African swine fever virus (ASFV) causes one of the most devastating diseases affecting pigs and wild suids, a worldwide epizootic situation exacerbated in recent years due to the lack of vaccine or effective treatment. ASFV has a restricted cell tropism, and is prone to replicate in porcine monocytes and alveolar macrophages with high efficiency. Here, the replication capabilities of ASFV were examined in swine pulmonary alveolar macrophages (PAMs) and compared with 3D4/21, PK-15, MA-104 and Marc-145 cell lines using PCR, qPCR and Western blot with monoclonal antibodies against the viral p30 and p72 proteins. The results showed that ASFV has a variety of infection characteristics in PAMs and showed four cell lines with distinct defects during virus early transcription-translation, genome replication and late protein synthesis. Furthermore, an antiviral role of the stress granule pathway was revealed against ASFV, and ASFV infection inhibited stress granule formation in PAMs but not 3D4/21. These results will help to deepen our knowledge on ASFV infection and to develop ASFV susceptible cell lines.

## 1. Introduction

African swine fever (ASF) is one of the most devastating diseases affecting pigs and wild suids and currently lacks either a vaccine or effective treatment [1]. ASF is endemic in sub-Saharan Africa [2], but since its introduction to the Caucasus region in 2007, a highly virulent strain of ASF virus (ASFV) has continued to circulate and spread into Eastern Europe and Russia, and most recently into Western Europe and Asia [3]. This is of particular concern in the case of China, producing half of the world’s pig population, where the disease was first reported in 2018 [4].

ASFV belongs to the family Asfarviridae, with a linear dsDNA genome of 170–194 kbp, encoding more than 150 polypeptides. The virus particle, of icosahedral morphology with a diameter of about 200 nm, contains more than 50 proteins. ASFV replication is limited to cells of the monocyte–macrophage lineage, and this tropism is thought to be crucial for disease pathogenesis [5,6]. The invasion of its host cell by a determined virus is a process that can be considered a sequence of successive steps, including virus binding, internalization, uncoating, early transcription-translation, genome replication, late protein synthesis and virus particle morphogenesis, to finally release the viral progeny [7]. Although early studies found that ASFV was able to enter a wide range of cell types from a number of different species in vitro, in most of these cells, the virus was unable to undergo a productive infection [8,9]. However, the mechanism underlying the limited cell tropism of ASFV and the block to virus replication in non-monocyte–macrophage cells is unknown due to a paucity of knowledge about ASFV–host interactions.

Stress granules (SG) are dynamically formed in the cytoplasm as a result of a viral infection, heat shock or treatment with chemical agents [10]. These structures affect mRNA stability in cells, block apoptosis and condition cell homeostasis [11]. Virus infection can certainly be considered a source of stress for cells, and several viruses have been studied to monitor their effect on the host stress response, especially how viruses modulate SG assembly. Several viruses have been reported to inhibit SG accumulation, such as human T cell leukemia virus type-1 (HTLV-1) [12], influenza A virus (IAV) [13] and herpes simplex virus 2 (HSV-1) [14]. In contrast, some viruses such as respiratory syncytial virus (RSV) and porcine reproductive and respiratory syndrome virus (PRRSV) induce the stable formation of SGs [15,16]. SGs, or at least SG components, may potentially function to affect productive viral infection. The propagation of some viruses, such as measles virus [17] and Japanese encephalitis virus [18] can be impaired by SGs or their components. Meanwhile, Chikungunya virus and Sindbis virus utilized the SG component G3BP2 as an essential part of viral replication [19,20]. Currently, the interplay between ASFV replication and the cellular stress response remains largely unclear.

Several cell lines, such as IPAM (immortalized swine pulmonary alveolar macrophage cell lines), COS-1 (monkey kidney tissue-derived cells), WSL (a wild boar lung cell line) and MA-104 (African green monkey kidney cell line) have been studied to propagate or titrate limited strains of ASFV [6,21,22,23,24]. However, most field-isolated ASFV strains showed deficient or partially deficient replication in those cell lines with unknown mechanisms. To explore the ASFV infection mechanisms, we used a prevalent ASFV genotype II strain isolated in China to investigate the replication of ASFV in five different cell lines originated from pig or monkey. Primary swine pulmonary alveolar macrophages (PAMs) are the natural target cells for ASFV; swine pulmonary alveolar macrophage cell line 3D4/21 is one clone of IPAM cells that was reported to support ASFV replication [23]; PK-15 is a swine kidney cell line with unknown susceptibility to ASFV; African green monkey kidney cell line MA-104 was identified as a suitable substrate for ASFV isolation [22], and its derivatives Marc-145 was also explored in this study. The variety of ASFV infection characteristics shown in the five detected cell lines helps to better understand the mechanisms of ASFV’s limited replication capabilities in cell lines and provides new insight for ASFV-susceptible cell line construction.

## 2. Materials and Methods

### 2.1. Viruses, Cells and Reagents

ASFV genotype II strain CN/GS/2018, which was isolated with PAMs at the Lanzhou Veterinary Research Institute, was used in the present study. The virus stock was the fifth passage cell culture prepared in PAMs with a titer of 10^6.47^ HAD_50_/mL. 3D4/21, PK-15, MA-104 and Marc-145 cells were purchased from American Type Culture Collection (ATCC). PAMs were collected from 3-week-old specific-pathogen-free (SPF) piglets that were free of ASFV, PRRSV, porcine circovirus type 2, pseudorabies virus, classical swine fever virus, porcine parvovirus, swine influenza virus and Mycoplasma hyopneumoniae infections using lung lavage as previously described [25]. In brief, the separated lungs from piglets were washed three times with RPMI-1640 medium (GIBCO, Thermo Scientific, Waltham, MA, USA). The washes were combined and centrifuged at 1500 rpm at 4 °C for 10 min. The cell pellets were resuspended and mixed with 20 mL of RPMI-1640 medium (GIBCO, Invitrogen Corporation, Carlsbad, CA, USA), 100 units/mL of penicillin and 100 μg/mL of streptomycin. The viability of PAMs determined by trypan blue dye exclusion was more than 95%. Cell concentration of the isolated PAMs was adjusted to 2.5 × 10^6^/mL with RPMI-1640 medium containing 10% fetal bovine serum (FBS) before seeding into 25 cm^2^ culture flasks (Costar, Corning Incorporated, Corning, NY, USA) and incubation for 12 h at 37 °C with 5% CO_2_ in a saturated humidified compartment to allow cells to adhere to flasks. This procedure yielded approximately 95% pure macrophages as determined by light microscopy. PK-15 and Marc-145 were maintained in Dulbecco’s modified Eagle’s medium (DMEM) (Gibco, Waltham, MA, USA) with 10% fetal bovine serum (FBS) (Lonsera, Canelones, Uruguay), penicillin (250 U/mL) and streptomycin (250 μg/mL); 3D4/21 were maintained in Roswell Park Memorial Institute (RPMI) 1640 with 20% FBS (Lonsera, Uruguay), penicillin (250 U/mL) and streptomycin (250 μg/mL); MA-104 were maintained in MEM with 10% FBS (Lonsera, Uruguay), penicillin (250 U/mL) and streptomycin (250 μg/mL). Detailed information about the cells is listed in Table 1.

### 2.2. Anti-ASFV p30/p72 Monoclonal Antibodies Preparation

ASFV p30 gene was amplified from ASFV genotype II strain CN/GS/2018 viral DNA with primers p30-F (5′-GCGGAATTCATGGATTTTATTTTAAATATA-3′) and p30-R (5′-CGCCTCGAGTTTTTTTTTTAAAAGTTTAAT-3′) and cloned into pET-32a (+) with *EcoRI* and *XhoI* restriction enzyme sites. The recombinant pET-32a-p30 was transformed into *E. coli* BL21(DE3) and cultured in Luria–Bertani liquid medium with 100 µg/mL of ampicillin. When the optical density at 600 nm of the culture reached 0.6, protein expression was induced with 1 mM isopropyl-β-D-1-thiogalactoside (IPTG) at 37 °C for 6 h. The recombinant p30 protein was expressed in inclusion body and purified by urea dialysis as previously described [26].

Partial ASFV p72 gene (1–987 bp) was amplified from ASFV genotype II strain CN/GS/2018 viral DNA with primers p72-F (5′-CCGGAATTCATGGCATCAGGAGGAG-3′) and p72-R (5′-CGCCTCGAGGAGCGCAAGAGGGGGC-3′) and cloned into pET-28a (+) with *EcoRI* and *XhoI* restriction enzyme sites. The recombinant pET-28a-p72 was transformed into *E. coli* BL21(DE3) and cultured in Luria–Bertani liquid medium with 100 µg/mL of kanamycin. When the optical density at 600 nm of the culture reached 0.6, protein expression was induced with 1 mM isopropyl-β-D-1-thiogalactoside (IPTG) at 37 °C for 6 h. The recombinant p72 protein was expressed in inclusion body and purified by urea dialysis as previously described [26].

Hybridoma technology was applied for preparation of anti-p30 and anti-p72 monoclonal antibodies (mAbs) as previously described [27].

### 2.3. Virus Passage Assay

Cells (the five selected cell lines) were infected with ASFV (multiplicity of infection (MOI) = 5) for 1 h at 37 °C and washed three times with PBS before incubation with fresh medium for another 47 h. At 48 h postinfection (hpi), the infected cells were freeze/thawed three times followed by centrifugation at 12,000× *g* for 5 min at 4 °C. Then the virus was collected (P1) and blind passaged onto fresh cells twice (P2 and P3). Viruses P1, P2 and P3 were collected for virus detection by PCR with primers targeting B646L gene (Table 2).

### 2.4. Virus Attachment Detection

Virus attachment capability was assessed indirectly through quantification of viral genomic DNA of the viruses that bound on the surface of the selected cells after wash off of the unbonded viruses within 1 hpi as described previously [28]. Cells were infected with ASFV (MOI = 5) for 15 min, 30 min and 1 h at 4 °C. The cells were then washed with ice-cold PBS three times, and the ASFV genomic DNA was extracted from the washed cells using E.A.N.A. Viral DNA Kit (Omega Bio-tek, Norcross, GA, USA) was utilized following the manufacturer’s instructions. The quantification of ASFV genomic DNA was performed with real-time PCR using AceQ qPCR Probe Master Mix (Vazyme, Shanghai, China) with primers targeting ASFV B646L gene (Table 2).

### 2.5. Virus Entry Detection

Virus entry capability was assessed indirectly through quantification of viral genomic DNA of the viruses that enters into the selected cells after wash off of noninternalized virus on the surface of the cells within 2 hpi as described previously [28]. Cells were infected with ASFV (MOI = 5), incubated for 1 h at 4 °C and washed with PBS 3 times before incubation for 30 min, 1 h and 2 h at 37 °C. The cells were washed with citrate buffer (pH = 3) to remove noninternalized virus. ASFV genomic DNA was extracted from the washed cells and quantified with real-time PCR as described above.

### 2.6. Virus Genomic DNA Replication Detection

Cells were infected with ASFV (MOI = 5) for 2 h at 37 °C and washed three times with PBS before incubation with fresh medium. Viral DNA was extracted from the infected cell lysates at 8, 24 and 48 hpi and quantified by real-time PCR as described above.

### 2.7. Viral Early and Late Genes Expressions Detection

Cells were infected with ASFV (MOI = 5) for 2 h at 37 °C, washed three times with PBS and incubated with fresh medium. The cells lysates were collected at 8, 24 and 48 hpi with 50 μL of RIPA lysis buffer (Beyotime, Shanghai, China) on ice for 30 min, then dissolved by SDS-PAGE and transferred onto a nitrocellulose membrane. After transfer, the membrane was incubated in blocking buffer (5% nonfat milk in PBST *w*/*v*) for 2 h at room temperature, washed five times with PBST, then tested with the following antibodies: anti-p30 monoclonal antibody (mAb) (1:2000) and anti-p72 mAb (1:2000), prepared in our laboratory; and anti-β-actin mAb (1:1000; Santa Cruz, CA, USA) at 4 °C overnight. The membranes were washed with PBST five times before incubation with HRP-conjugated goat anti-mouse IgG (H-L) secondary antibody (1:1000; Beyotime, China) for 1 h at room temperature. Bound proteins were exposed with an ECL Kit (Tanon, Shanghai, China) after secondary antibody incubation and an additional five washes with PBST.

### 2.8. Viral Genes Transcription Quantification

PAMs and 3D4/21 cells were infected with ASFV (MOI = 5) for 2 h at 37 °C and washed three times with PBS before incubation with fresh medium. Total RNAs were extracted from the infected cells at 8, 24 and 48 hpi using Total RNA Kit (Omega Bio-tek, USA) following the manufacturer’s instructions. ASFV CP204L mRNA was detected using HiScript II One Step qRT-PCR SYBR Green Kit (Vazyme, China), and the detection of B646L mRNA was performed using HiScript II One Step qRT-PCR probe kit. The primers and probes used in this study are listed in Table 2. Each reaction was performed in triplicate, and results are expressed as mean ± standard deviation (SD).

### 2.9. Virus Assembly Detection

PAMs, MA-104 and Marc-145 cells were infected with ASFV (MOI = 5) for 2 h at 37 °C and washed three times with PBS before incubation with fresh medium. At 48 hpi, the supernatant was removed, and the cells were washed three times with PBS. The cells were then freeze/thawed three times with 500 μL PBS, and supernatant was collected after centrifugation at 12,000× *g* for 5 min at 4 °C. The viral genomic DNA was quantified using qPCR, and infectious virus particles were detected and titrated by hemadsorption assay. Swine PBMC were isolated using Pig Peripheral Blood Lymphocyte Separation Solution KIT (Solarbio, Beijing, China) following the instructions, and the isolated cells were plated into 96-well plates the night before. The virus was serially tenfold diluted from 10^0^ to 10^−8^ with RPMI 1640 medium and added into the PBMC in 96-well plates in six replicates. After 2 h of infection, the supernatant was removed and replaced with fresh RPMI 1640 medium with 10% FBS, and cells were incubated at 37 °C with 5% CO_2_. At 24 hpi, swine red blood cells were added into the PBMCs for 6 days of incubation at 37 °C with 5% CO_2_. Hemadsorption was observed and HAD_50_ was calculated for virus titration.

### 2.10. Virus Release Detection

PAMs, MA-104 and Marc-145 cells were infected with ASFV (MOI = 5) for 2 h at 37 °C and washed three times with PBS before incubation with fresh medium. At 48 hpi, both supernatant and cells were collected for virus titration by hemadsorption assay as described above.

### 2.11. Indirect Immunofluorescence Assay

Cells were infected with ASFV (MOI = 5) for 2 h at 37 °C and washed three times with PBS before incubation with fresh medium. At 23 hpi, the infected cells were transferred into a 50 °C incubator for 20 min and fixed with 4% paraformaldehyde (PFA) at room temperature (RT) for 30 min. The fixed cells were then washed with PBS 5 times and permeabilized with methanol for 10 min at −20 °C, followed by another 5 times of washing. Then the cells were blocked with 2% BSA PBS at room temperature for 2 h and incubated with mouse anti-p30 mAb and rabbit anti-G3BP2 antibody (1:200, abcam) at 4 °C overnight. After washing, the cells were incubated with Coralite488-conjugated Affinipure goat anti-mouse IgG(H + L) and Coralite594-conjugated goat anti-rabbit IgG(H + L) for 1 h at 37 °C and stained with DAPI for 5 min at RT. The fluorescence was examined with a Zeiss Axio Observer and quantified with ImageJ (at least 30 cells from 5 different fields of view were analyzed for quantification).

### 2.12. SG Effect on ASFV Replication

PAMs were infected with ASFV (MOI = 5) for 2 h at 37 °C and washed three times with PBS before incubation with fresh medium. Cells were heat-shocked for 20 min at 50 °C or not treated before collected at 24 hpi for detection by Western blot with anti-eIF2α antibody (1:2000, Cell Signaling Technology, Danvers, MA, USA) and anti-Phospho-eIF2α (Ser51) antibody (1:2000, Cell Signaling Technology) as described above.

SiRNAs used to knock down G3BP1 (si-G3BP1: CAUUAACAGUGGUGGGAAA) and G3BP2 (si-G3BP2: GGAGCAAGAAGAAAGACAA) were designed and synthesized by Biotend, Shanghai. 50 nM si-G3BP1 and 50 nM si-G3BP2 or 100 nM negative control siRNA (si-nc) were transfected into 3D4/21 cells using lipfectamine 3000 (Invitrogen) according to the instructions for 12 h before subsequent ASFV infection at 5 MOI. Cells were collected at 48 hpi for detection by western blot with anti-G3BP1 antibody (1:2000, GeneTex, Irvine, CA, USA) and anti-G3BP2 antibody (1:2000, Abcam, Cambridge, UK) as described above.

### 2.13. Biosafety Statement and Facilities

All experiments with live ASFV in this study were performed in the biosafety level 3 lab at the Lanzhou Veterinary Research Institute.

## 3. Results

### 3.1. ASFV Passage in Different Cells

**Figure 1:**
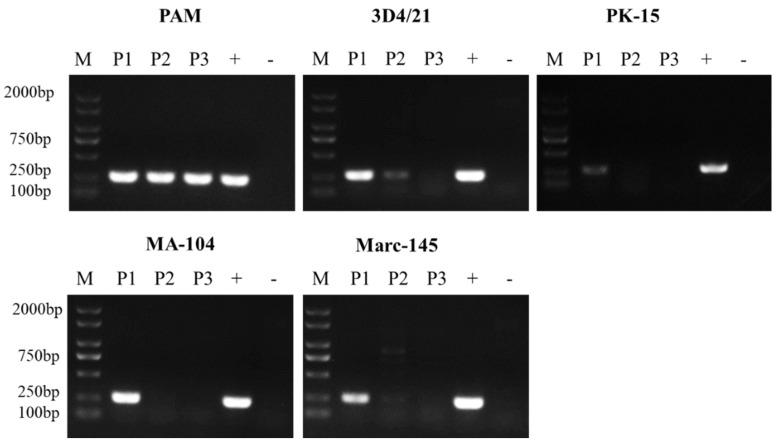
ASFV genomic DNA detection in PAMs, 3D4/21, PK-15, MA-104 and Marc-145 cells. The cells were infected with ASFV at 5 MOI. Virus culture supernatants (P1) were collected at 48 hpi and blind passaged twice (P2 and P3). The P1–3 viruses were detected with PCR with primers for the viral B646L gene. +: PCR positive control. -: PCR negative control.

ASFV obtained from PAMs was used to infect the five cell lines and blind passaged twice. The virus stocks from each passage were collected and analyzed with PCR for the B646L gene. As shown in Figure 1, ASFV grows consistently and stably in PAMs, but it exhibits a growth defect in 3D4/21, MA-104 and Marc-145 cells, as shown by a progressive loss of PCR positivity. This defect was more marked in PK-15 cells, with very weak PCR positivity even after the first passage of virus (P1).

### 3.2. ASFV Attachment on Different Cells

**Figure 2:**
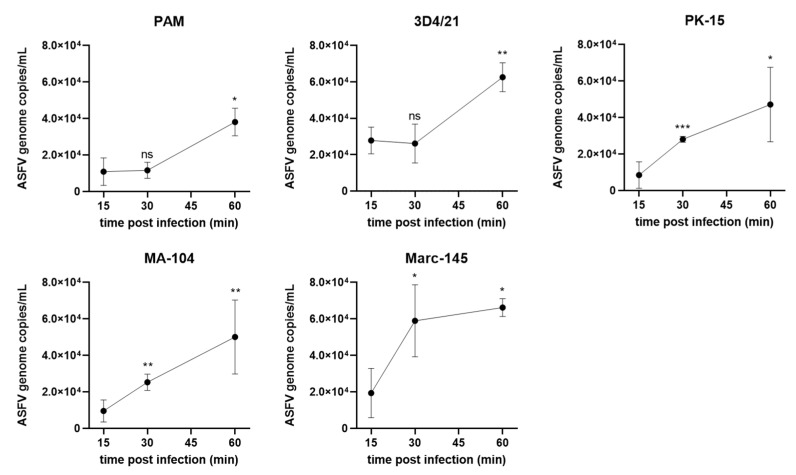
ASFV genomic DNA quantification after attachment on PAMs, 3D4/21, PK-15, MA-104 and Marc-145 cells. The cells were infected with ASFV at 5 MOI and incubated at 4 °C for 15 min, 30 min and 1 h for detection of virus attachment. ASFV genomic DNA was quantified with real-time PCR with primers targeting B646L gene. Results are presented as the mean ± SD of data from three independent experiments; ns, not significant; *, *p* < 0.05; **, *p* < 0.01, ***, *p* < 0.005.

PAMs and the 4 cells lines were infected with ASFV at 5 MOI and incubated for 15 min, 30 min and 1 h at 4 °C. Virus attachment was detected with qPCR as shown in Figure 2. The results showed that ASFV was able to attach to all the five cell lines at a similar level. At 15 min post infection (mpi), the viral genomic DNA copies detected in the five different cell lines were similar to each other and continued to increase modestly up to 5~6 × 10^4^ copies/mL at 60 mpi.

### 3.3. ASFV Entry into in Different Cells

**Figure 3:**
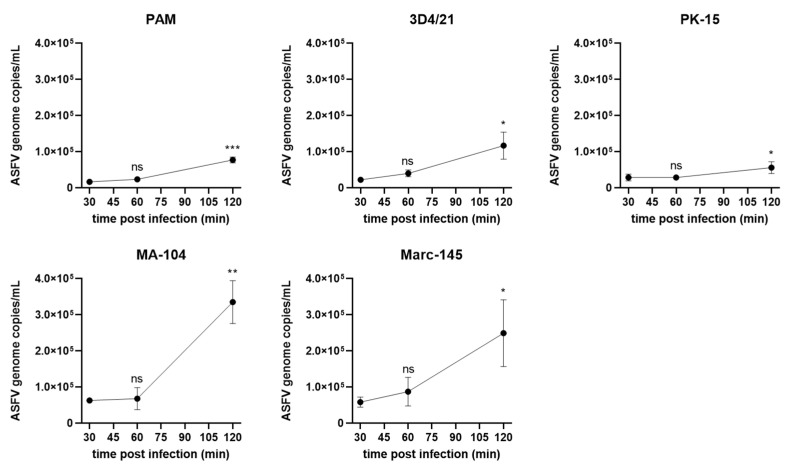
ASFV genomic DNA quantification after entry into PAMs, 3D4/21, PK-15, MA-104 and Marc-145 cells. The cells were infected with ASFV at 5 MOI and incubated for 1 h at 4 °C. After washing with PBS 3 times and incubation for 30 min, 1 h and 2 h at 37 °C, ASFV genomic DNA was quantified with real-time PCR with primers targeting B646L gene. Results are presented as the mean ± SD of data from three independent experiments; ns, not significant; *, *p* < 0.05; **, *p* < 0.01, ***, *p* < 0.005.

PAMs and the 4 cell lines were infected with ASFV (MOI = 5), incubated for 1 h at 4 °C and washed with PBS 3 times before incubation for 30 min, 1 h and 2 h at 37 °C. The cells were washed with citrate buffer (pH = 3) to remove noninternalized virus before viral genomic DNA was extracted and quantified (Figure 3). The levels of the viral genomic DNA in PAMs, 3D4/21, MA-104 and Marc-145 cells increased by 1.5~4.3-fold from 30 mpi to 120 mpi, indicating that virus particles had entered into the cells. PK-15 cells only exhibited a low but still significant increase in intracellular viral genomic DNA from 30 mpi to 120 mpi, indicating that ASFV virions entered into PK-15 cells with low efficiency. Interestingly, much more viral genomic DNA was detected in MA-104 and Marc-145 cells in comparison with PAMs within the 120 mpt. Although the same number of cells and the same viral loads were used for each cell line, PAMs are smaller than MA-104 and Marc-145 cells, and therefore it was possible that the larger surface area of MA-104 and Marc-145 cells provided more opportunities for virus entry.

### 3.4. ASFV Genome Replication in Different Cells

**Figure 4:**
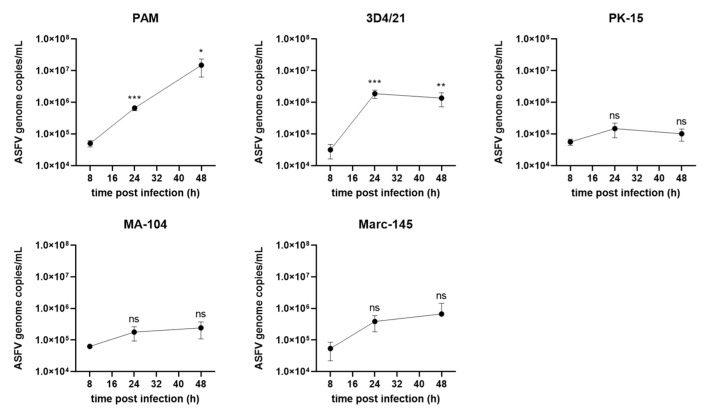
ASFV genomic DNA quantification during genome replication in PAMs, 3D4/21, PK-15, MA-104 and Marc-145 cells. The cells were infected with ASFV at 5 MOI for 2 h at 37 °C. After washing three times with PBS, and incubation with fresh medium, the viral DNA was extracted from the infected cells at 8, 24 and 48 hpi and quantified by real-time PCR with primers targeting B646L gene. Results are presented as the mean ± SD of data from three independent experiments; ns, not significant; *, *p* < 0.05; **, *p* < 0.01, ***, *p* < 0.005.

PAMs and 4 cell lines were infected with ASFV at 5 MOI and the viral genomic DNA was detected by qPCR at 8, 24 and 48 hpi. As shown in Figure 4, in PAMs, a consistent increase in ASFV genomic DNA levels was observed during virus infection. In 3D4/21 cells viral genomic DNA levels peaked at 1.9 × 10^6^ copies/mL at 24 hpi, 8-fold lower than in PAMs. The viral genomic DNA in PK-15 cells at 24 or 48 hpi showed no significant increase compared with that at 8 hpi, indicating a defect of genome replication of ASFV in PK-15 cells. In MA-104 and Marc-145 cells, although the ASFV genome replication displayed a similar trend as that in PAMs, the viral genomic DNA copies in MA-104 and Marc-145 cells were about 62-fold and 22-fold lower than those in PAMs, respectively. These data suggest that PAMs supported the highest levels of ASFV genomic replication.

### 3.5. ASFV Early and Late Gene Expression in Different Cells

**Figure 5:**
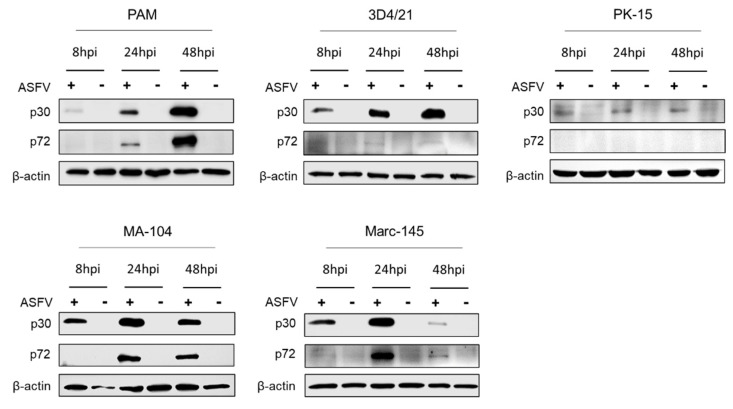
ASFV early and late genes expressions in PAMs, 3D4/21, PK-15, MA-104 and Marc-145 cells. The cells were infected with ASFV at 5 MOI for 2 h at 37 °C. After washing with PBS three times, and incubation with fresh medium, the cells lysates were collected at 8, 24 and 48 hpi, and ASFV p30 and p72 protein expressions were detected by western blot. +: ASFV infected; -: mock.

As shown Figure 5, in PAMs, levels of the early gene product p30 increased from 8 to 48 hpi, whereas (as expected) the late gene product p72 started to be detected from 24 hpi and increased at 48 hpi. In comparison with PAMs, both p30 and p72 expression levels in MA-104 and Marc-145 cells reached the highest levels at 24 hpi and then sharply declined. In 3D4/21 cells only p30 could be detected from 8 hpi and increased up to 48 hpi. Additionally, p72 could hardly be detected by western blot. In addition, low levels of p30 and no p72 could be detected in PK-15 cells infected with ASFV. As early gene expression proceeded before viral genomic DNA replication, the low level of viral p30 protein might explain the low-level genome replication of ASFV in PK-15 cells as shown in Figure 4.

### 3.6. ASFV Early and Late Genes Transcription in 3D4/21 Cells

**Figure 6:**
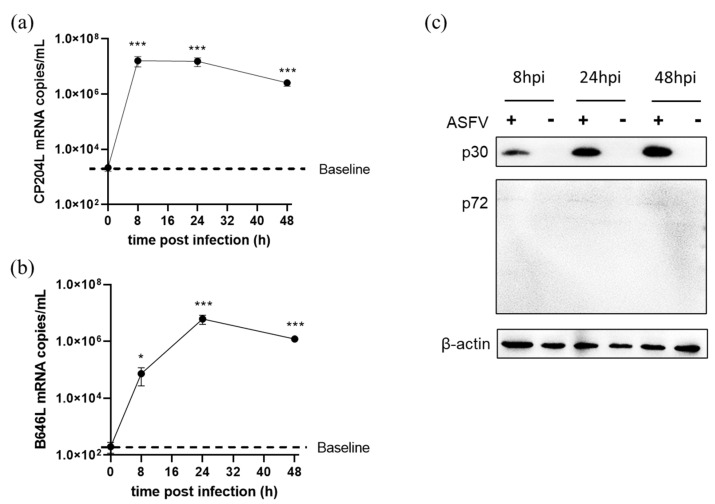
ASFV early and late genes transcription in 3D4/21 cells. The cells were infected with ASFV at 5 MOI for 2 h at 37 °C. After washing with PBS three times and incubation with fresh medium, the total RNAs were extracted from the infected cells at 8, 24 and 48 hpi and used for quantification with real-time RT-PCR. (**a**) Quantification of ASFV early gene CP204L mRNA. (**b**) Quantification of ASFV late gene B646L mRNA. (**c**) Expressions of the viral p30 and p72 proteins in ASFV-infected 3D4/21 cells detected by western blot. *, *p* < 0.05; ***, *p* < 0.005. +: ASFV infected; -: mock.

In order to explore whether the block to p72 protein expression occurred during gene transcription or translation in 3D4/21 cells, the cells were infected with ASFV and harvested at 0, 8, 24 and 48 hpi for RNA extraction. qRT-PCR results showed that CP204L (p30) mRNA increased rapidly from 0 to 8 hpi and remained at a high level until 48 hpi (Figure 6a). Meanwhile, B646L mRNA levels increased up to 6.11 × 10^6^ copies/mL at 24 hpi (Figure 6b). Further, western blot results showed that p30 expression levels also increased from 8 to 48 hpi, but p72 protein could only just be detected during the viral infection course (Figure 6c), suggesting a defect of late protein translation during ASFV infection in 3D4/21 cells.

### 3.7. ASFV Infectious Virions Production and Release in PAMs, MA-104 and Marc-145 Cells

**Figure 7:**
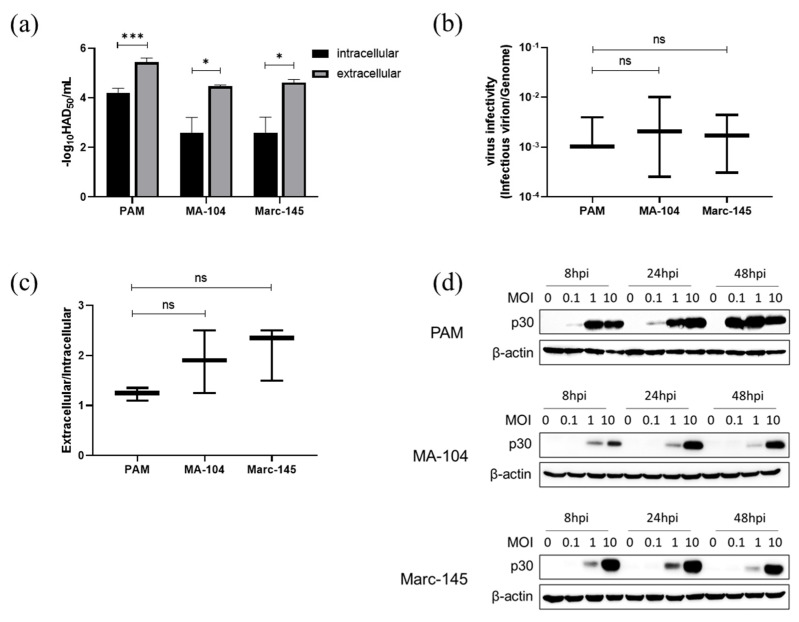
ASFV assembly and release in PAMs, MA-104 and Marc-145 cells. Cells were infected with 5 MOI ASFV for 2 h at 37 °C and washed three times with PBS and incubated with fresh medium for 48 h. The cell supernatant and cells were collected, respectively, for virus titration and genomic DNA quantification. (**a**) Intra- and extracellular virus titers in PAMs, MA-104 and Marc-145 cells. (**b**) Graphical representation of the ratio of infectivity to genomic DNA. (**c**) Graphical representation of the ratio of extracellular to intracellular virus titres. (**d**) The viral p30 protein in cells infected with ASFV at different MOIs was detected with western blot at the indicated 3 time points. ns: no significant difference by *t*-test. *, *p* < 0.05; ***, *p* < 0.005.

As ASFV genome replication and viral protein expression were detectable in MA-104 and Marc-145 cells but virus could not be serially passaged in these cells, infectious virions produced in ASFV-infected PAMs, MA-104 and Marc-145 cells were then examined. As shown in Figure 7a, both MA-104 and Marc-145 cells inoculated with 5 MOI ASFV were able to produce and release infectious ASFV virions. The similar ratios of infectious virions/genome DNA (48 hpi) among MA-104, Marc-145 and PAMs indicated a sufficient package capability of ASFV in MA-104 and Marc-145 (Figure 7b). The ratios of extracellular/intracellular virus showed no significant differences between the virus in PAMs and MA-104 or Marc-145 cells (Figure 7c). It suggests that ASFV could complete a replication cycle in MA-104 and Marc-145 cells. However, the detection of ASFV infection at different MOIs showed that the virus replication in MA-104 or Marc-145 cells could only be detected when MOI ≥ 1 (Figure 7d).

### 3.8. Role of SG Formation during ASFV Replication

**Figure 8:**
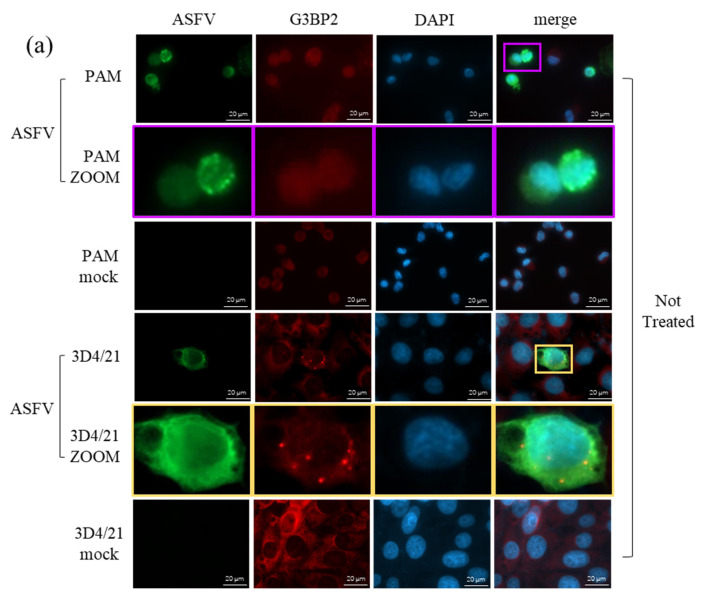

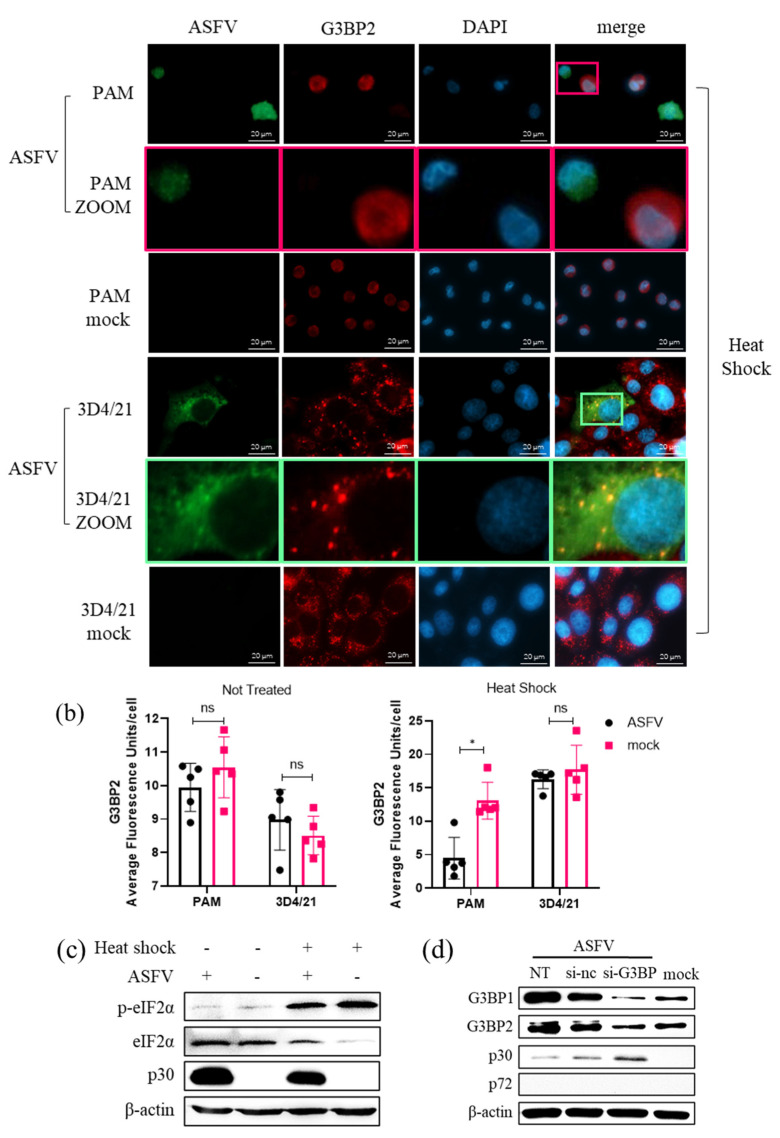
SG formation in the different cells. (**a**) The cells were infected with ASFV at 5 MOI and heat-shocked for 20 min at 50 °C or not treated before fixation at 24 hpi with 4% PFA. ASFV was labeled by p30 protein, SG formation was labeled by G3BP2. Colored squares indicate corresponding zoom views. (**b**) G3BP2 fluorescence intensity in ASFV-infected or mock cells quantified with ImageJ. ns: no significant difference by *t*-test; *, *p* < 0.05. (**c**) Effect of SG formation on ASFV infection in PAMs. PAMs were infected with ASFV at 5 MOI and collected at 24 hpi after heat shock or no treatment. Phosphorylation of eIF2α and viral p30 protein was detected by western blot. (**d**) Effect of SG formation on ASFV infection in 3D4/21 cells. 3D4/21 cells were transfected with siRNAs against G3BP1/2 (si-G3BP), negative control (si-nc) or not transfected (NT) for 12 h before infected with ASFV at 5 MOI and collected at 48 hpi. Expression of G3BP1, G3BP2, viral p30 and p72 proteins was detected by western blot.

The cellular stress-induced complex SG is an important regulator of cellular protein translation, and it has been proved to be involved during many viruses’ infection. The previous results in this study showed that ASFV late protein translation was suppressed in 3D4/21 cells; therefore we next explored the role of SG during ASFV replication. PAMs and 3D4/21 cells were infected with ASFV and incubated for 24 h at 37 °C, or for 24 h at 37 °C and another 20 min at 50 °C, and then SG formation was detected by indirect fluorescence assay (IFA). As shown in Figure 8a, typical SG dots formed in ASFV-infected 3D4/21 cells, but not in mock or PAMs inoculated with ASFV. Meanwhile, the heat-induced G3BP2 signal in ASFV-infected PAMs were significantly lower than those in mock cells, but the heat-induced SG formed in both ASFV- and mock-infected 3D4/21 cells with no significant differences (Figure 8a,b). These results indicated that ASFV infection in 3D4/21 cells induced SG formation while in PAMs, ASFV infection suppressed G3BP2 expression, andthus the SG formation. SG formation in MA-104 and Marc-145 was also detected, and the results showed that ASFV infection in MA-104 and Marc-145 could also suppress heat-shock-induced SG formation (Figures S1 and S2).

To explore the effect of SG formation on ASFV infection, PAMs were infected with ASFV and incubated for 24 h, follow by 20 min incubation at 50 °C; eIF2α phosphorylation and the viral p30 were detected by western blot. The result showed that eIF2α was highly phosphorylated, indicating the activation of the SG pathway, and the viral p30 production decreased after heat shock, indicating that heat-shock-activated SG pathway inhibited ASFV replication (Figure 8c). In 3D4/21 cells, both G3BP1 and G3BP2 were knocked down by siRNAs to restrain SG formation and infected with ASFV. As shown in Figure 8d, in comparison between the ASFV/NT (not transfected) group and the mock group, ASFV infection led to an increase in G3BP1/2 expression, and thus the SG formation (consistent with that shown in Figure 8a). Moreover, in the si-G3BP group, the expression of ASFV p30 increased in comparison with the NT and si-nc groups, indicating that SG formation has an antiviral role. These results gave a hint that the SG pathway played an antiviral role during ASFV infection in both PAMs and 3D4/21 cells. However, p72 expression could still not be detected in 3D4/21 cells with impaired G3BP1/2 proteins, indicating that the defect of ASFV late protein translation in 3D4/21 cells might not be due to the SG antiviral pathway.

## 4. Discussion

ASFV isolates derived from pigs have a restricted cell tropism and replicate only in porcine monocytes and alveolar macrophages. Therefore, primary monocytes or alveolar macrophages have been used to study the virus–host interaction and mimic ASFV infections in vivo [6]. However, working with primary cells also has some disadvantages, including low reproductivity, high batch-to-batch variation, high time consumption, expensive extraction and animal welfare considerations [6]. In addition, it is also difficult to perform gene manipulation in primary cells such as knock-out or over-expression to study the virus–host interactions. It is necessary to find or develop a cell line suitable for high-productive ASFV replication. In this study, ASFV replication was explored in PAMs and four cell lines. The results showed that ASFV grew well in PAMs but not in PK-15 cells. MA-104 and Marc-145 cells were susceptible to ASFV infection only when inoculated with high doses of virus. An abortive infection of ASFV was shown in 3D4/21 cells as ASFV was able to attach and enter into the cells, express early viral proteins and proceed to genome replication but could not produce infectious virions due to its defect in late protein translation. In addition, it was found that the SG pathway played antiviral roles during ASFV infection.

The ASFV infection cycle starts with viral adsorption and entry into the host cell and reaches the endocytic pathway [29] before being transported by microtubules to their replication site in the perinuclear area close to the microtubule organizing center (MTOC) [30]. The genomes of ASFV vary in length between 170 and 190 kbp and encode between 151 and 167 open reading frames. The transcription of viral genes is strongly regulated. Four classes of mRNAs have been identified by their distinctive accumulation kinetics-including immediate-early, early, intermediate, and late transcripts. Immediate early and early genes, such as p30 protein, pG1340L protein and helicase pD1133L, are expressed before the onset of DNA replication due to the action of several enzymes involved in the viral transcription that are packed in the viral core [31,32]. Following DNA replication, the transcription of intermediate and late genes begins such as the major capsid p72 protein, p11.5 protein, pH339R protein [31,32]. After viral proteins and DNA are accumulated and newly synthesized virions are assembled in the viral factory, the mature particle is transported to the cell surface through a microtubule-mediated mechanism [33] and budding at the membrane, acquiring an additional envelope [34]. Both intracellular and extracellular viruses are infectious but structurally and antigenically different [35]. In the present study, 3D4/21 cells were used to inoculate with ASFV, and western blot results showed that the viral early gene product p30 was obviously expressed while the viral late gene product p72 was not expressed. However, qRT-PCR results showed that both CP204L and B646L mRNA levels significantly increased rapidly at 8 hpi and remained at a high level till 24–48 hpi. It suggested that the ASFV infection is blocked at the late protein translation stage in 3D4/21 cells. One important pathway known to regulate protein translation in cells post a variety of stresses such as virus infection is the SG pathway. The dsRNA produced during virus replication generally induces SG formation and the relevant antiviral processes in cells. Herpes simplex virus (HSV) virion host shutoff protein (vhs) has been proved to inhibit SG formation and boost viral true late gene production in a cell-type-dependent manner, and the HSV-Δvhs mutant infection induced cellular SG formation and a defect in viral late gene expression [14,36]. It suggests that the SG pathway plays a significant role in late protein expression during some virus infections. In this study, we observed that the SG pathway played an antiviral role against ASFV replication, and ASFV inhibited SG formation in PAMs but not in 3D4/21 cells. Further exploration showed that the knockdown of G3BP1/2 protein in 3D4/21 cells did not retrieve the expression of p72 protein, suggesting that SG might not be the determinant in the defect of p72 expression in 3D4/21 cells. Otherwise, it is possible that SG was still formed with the residual G3BP proteins and that was enough for inhibiting ASFV late protein translation. The underlying mechanism should be further studied in the future.

ASFV replicates mainly in various macrophage populations of spleen, lymph node, lung, liver and kidney [37,38,39]. Among various types of macrophages assessed, PAMs were suggested as they were more susceptible to ASFV infection in comparison with bone marrow-derived macrophages or blood monocytes. MA-104 is a cell line isolated from Cercopithecus aethiops, and kidney epithelial cells, commonly used for PRRSV diagnosis and for simian rotavirus production, and Marc-145 cells is one of its derivatives. In this study, our results showed that ASFV was able to complete the replication cycle, producing and releasing infectious virions in MA-104 and Marc-145 cells. However, it was interesting to find that ASFV was not able to be passaged in these two cell lines. Virus titration by hemadsorption assay in PBMCs indicated that intact, infectious virions were produced in MA-104 and Marc-145 cells. Further exploration indicated that MA-104 and Marc-145 cells were susceptible to virus infection only when inoculated with high doses of ASFV obtained from PAMs. This experiment should be repeated with high doses of ASFV obtained from MA-104 and Marc-145 cells and concentrated by ultracentrifuge in the future. As ASFV has been reported to enter cells through different pathways such as endocytosis [7] or micropinocytosis [40], we supposed that the different ways ASFV utilized to enter PAMs or MA-104/Marc-145 cells might determine the virus infection efficiency. The assessment of virus attachment and entry process through electron microscopy with purified virions will help to better understand the differences between PAMs and MA-104/Marc-145 cells during ASFV infection, which still needs further exploration in the future. Moreover, the ASFV genome replication capability in MA-104 and Marc-145 cells was lower than that in PAMs, possibly due to some unknown virus–host interactions.

Meanwhile, ASFV could not grow well in PK-15 cells as the viruses attached on the cells with high efficiency and then followed a sluggish entry process. The early gene product p30 was expressed at an extremely low level, and no significant genome replication could be detected. The underlying mechanism still needs further study.

All 5 cell types detected in this study displayed a sensitivity to ASFV attachment and entry, which was consistent with the previous studies showing that ASFV was able to bind and internalize into 16 cell lines derived from different species [9], indicating that ASFV has a wide cell tropism. However, as the ASFV used to assess the virus attachment and entry capabilities in this study was produced in PAMs, there is a possibility that the cellular proteins from PAMs played a role during ASFV attachment and entry into the different stable cell lines. Purified virions could be used to verify this, which was not performed in this study due to the lack of facilities in our BSL3. Additionally, so far, no cell line has been reported to be totally not infected, even when not attached to ASFV. Therefore, a perfect negative control that esd not attached by ASFV was not used in this study. Further study on this issue is under exploration.

In conclusion, in this study, our results showed that a genotype II ASFV strain could grow in PAMs, MA-104 and Marc-145 cells but not PK-15 cells, and it showed an abortive infection in 3D4/21 cells due to its defect in ASFV late protein translation. In addition, it first found the antiviral function of SG pathway against this virus, and ASFV infection in different cells developed various evasions in response. This should be helpful for better understanding ASFV infection mechanisms and developing ASFV susceptible cell lines.

## Figures and Tables

**Table 1:** Cells used to detect ASFV replication.

Cell	Organism	Tissue	Cell Type
PAM	Sus scrofa	Lung	Primary macrophage, alveolar
3D4/21	Sus scrofa	Lung	Macrophage, alveolar
PK-15	Sus scrofa	Kidney	epithelial
MA-104	Cercopithecus aethiops	Kidney	epithelial
Marc-145	Cercopithecus aethiops	Kidney	epithelial

**Table 2:** Primers used for ASFV detection.

Target Detection	Primers	Sequences
PCR	B646L-F	5′-AGTTATGGGAAACCCGACCC-3′
	B646L-R	5′-CCCTGAATCGGAGCATCCT-3′
qPCR for ASFV genomic DNA /RT-qPCR for B646L mRNA	B646L-qPCR-F	5′-CGGAGAACGACTTTATGA-3′
	B646L-qPCR-R	5′-CCTGGGATGCAAAATTTG-3′
	B646L-qPCR-probe	FAM-CAAGCGTTGTGACATCCGAACTATATT-TAMRA
RT-qPCR for CP204L mRNA	CP204L-qPCR-F	5′-AGGAGACGGAATCCTCAGCA-3′
	CP204L-qPCR-R	5′-GGGCTCTTGCTCAAACAACG-3′

## Data Availability

Not applicable.

## Supplementary Materials

The following supporting information can be downloaded at: https://www.mdpi.com/article/10.3390/v14122642/s1, Figure S1. SG formation in the MA-104 cells. MA-104 cells were infected with ASFV at 5 MOI and heat-shocked for 20 min at 50 °C or not treated before fixation at 24 hpi with 4% PFA. ASFV was labeled by p30 protein; SG formation was labeled by G3BP2. Figure S2. SG formation in the Marc-145 cells. Marc-145 cells were infected with ASFV at 5 MOI and heat-shocked for 20 min at 50 °C or not treated before fixation at 24 hpi with 4% PFA. ASFV was labeled by p30 protein; SG formation was labeled by G3BP2.
