# Supplementary material for: African Swine Fever Virus Exhibits Distinct Replication Defects in Different Cell Types
Source: Viruses. 2022 Nov 26;14(12):2642. doi: 10.3390/v14122642 (PMC9781062; doi:10.3390/v14122642)
Supplement: Supplementary file 1 [file viruses-14-02642-s001.zip › viruses-2054735-supplementary.pdf]

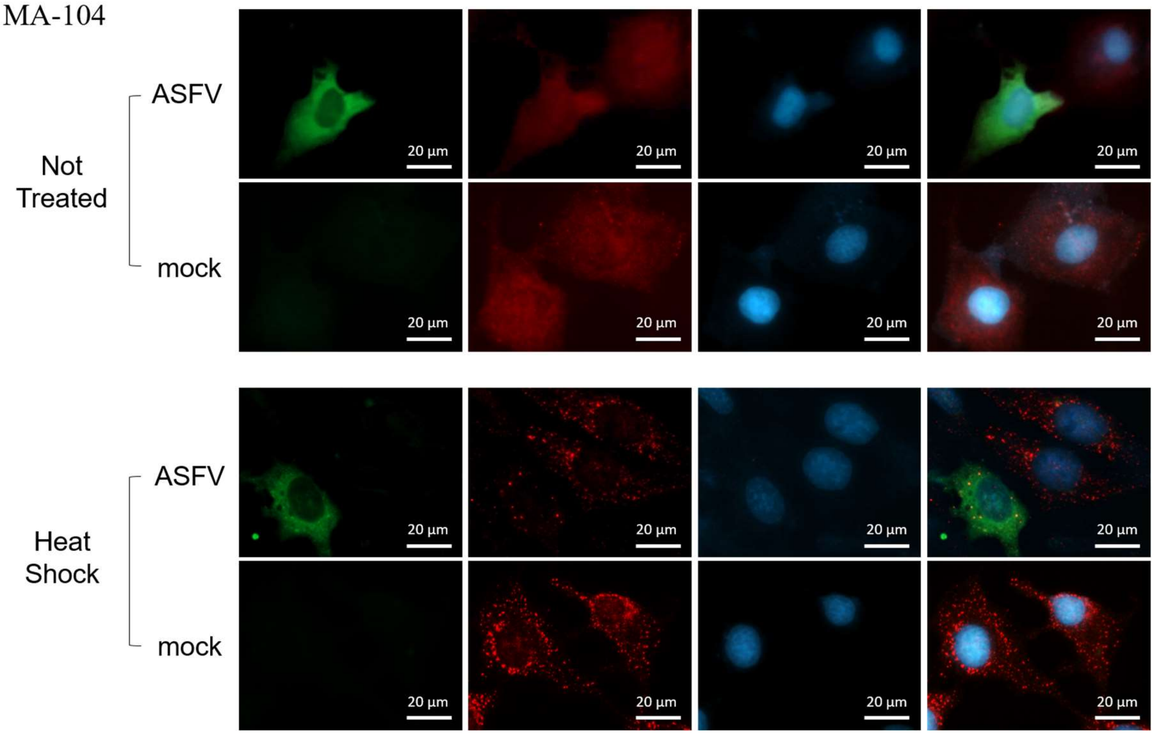

Figure S1. SG formation in the MA−104 cells. MA−104 cells were infected with ASFV at 5 MOI and heat−shocked for 20 min at 50 °C or not treated before fixation at 24 hpi with 4% PFA. ASFV was labeled by p30 protein; SG formation was labeled by G3BP2.

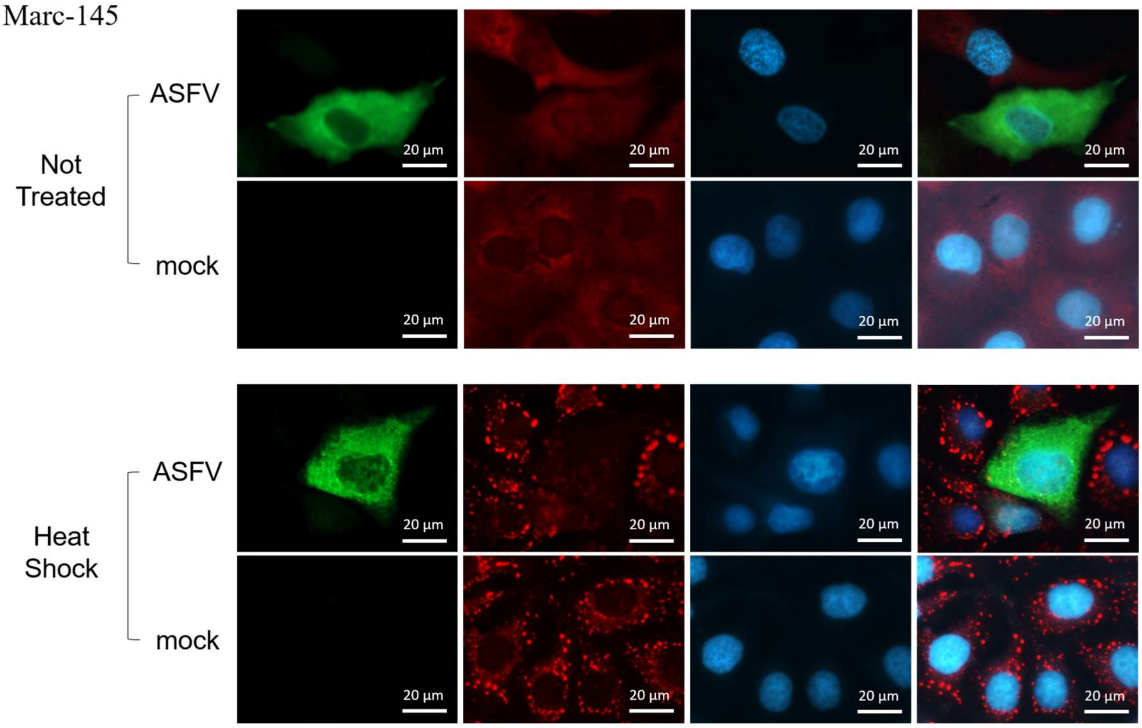

Figure S2. SG formation in the Marc−145 cells. Marc−145 cells were infected with ASFV at 5 MOI and heat−shocked for 20 min at 50 °C or not treated before fixation at 24 hpi with 4% PFA. ASFV was labeled by p30 protein; SG formation was labeled by G3BP2.
